# Tibial Bone Transcortical Erosion Caused by a Posterior Tibial Artery Pseudoaneurysm: A Rare Case Report

**DOI:** 10.1155/crra/1862171

**Published:** 2026-06-08

**Authors:** Abdudin Heru Mehammed, Muluken Yifru Gebresilassie, Seid Mohammed Shobe, Hawi Dida Midekso, Mohammed Abdulahi, Yididiya Getu Gebre, Bethelhem Belachew

**Affiliations:** ^1^ Department of Radiology, St. Paul′s Hospital Millennium Medical College, Addis Ababa, Ethiopia; ^2^ Department of Radiology, Addis Ababa University, Addis Ababa, Ethiopia, aau.edu.et

**Keywords:** aneurysm, posterior tibial artery, pseudoaneurysm

## Abstract

Pseudoaneurysm is an abnormal outpouching from the arterial wall containing only the outermost adventitial layer. As compared with this, true aneurysms contain all three layers: intima, media, and adventitia. Pseudoaneurysms are formed when blood traverses through the intima and media but is held by an adventitial layer. There are several etiologies for the formation of pseudoaneurysms, the most common one being posttraumatic laceration or dissection of the vessel wall. Postprocedural iatrogenic injury during surgery or biopsy is also another common cause for the development of pseudoaneurysms. Vasculitis, regional inflammatory conditions (like pancreatitis causing splenic pseudoaneurysm), and penetrating atheromatous ulcer are among the less common causes. Here, we present a case of a chronic posterior tibial artery pseudoaneurysm following a remote gunshot injury that resulted in extensive transcortical erosion with intraosseous extension and tibial bone transcortical erosion on a 33‐year‐old male patient with no known prior medical illness. Multimodal imaging including Doppler ultrasound, MRI (with contrast), and CT angiography established the diagnosis after an initial fine‐needle aspiration (FNA) attempt led to significant hemorrhage. Only a few cases have been reported on pseudoaneurysms of this size and tibia bone erosion. To our knowledge, full tibial transcortical erosion caused by a chronic posterior tibial artery pseudoaneurysm has not been clearly documented in the literature. This case highlights the importance of early vascular imaging prior to biopsy of pulsatile or indeterminate soft tissue masses.

## 1. Introduction

An aneurysm is a localized permanent dilatation of a blood vessel with at least a 50% increase of its expected normal caliber. Based on the involvement of blood vessel wall layers, there are two types of aneurysms: true and false aneurysms [1].

Pseudoaneurysms are contained arterial wall disruptions where blood escapes through a defect in the intima and media and is confined by adventitia or surrounding soft tissues. In contrast, a true aneurysm is bounded by all the three layers of the vessel wall. Pseudoaneurysms commonly develop following direct arterial damage, which may be caused by medical procedures (iatrogenic) or external trauma [2–6]. Alternatively, they can arise indirectly from inflammatory processes that erode the vessel wall and connective tissue disorders [7].

Pseudoaneurysm typically presents clinically as a pulsatile mass, often accompanied by an audible bruit [8]. On imaging evaluation, ultrasound, which is the first‐line modality, characteristically demonstrates the “yin–yang” sign on color Doppler and a to‐and‐fro waveform on spectral analysis. CT angiography (CTA) and magnetic resonance imaging (MRI) are additionally utilized for more detailed assessment, particularly to delineate the extent of the lesion and its relationship to adjacent structures [3, 9].

Because of their deep intermuscular location and smaller size, posterior tibial artery pseudoaneurysms are rare [2–5, 10]. Not only the location, but the presence of chronic evolution of pseudoaneurysm leading to extensive tibial cortical destruction and full transcortical erosion into the medullary cavity and anterior subcutaneous tissues makes this case extremely uncommon.

Using PubMed, Scopus, and Google Scholar a focused literature search was conducted between January 2000 and February 2026 using the terms: “posterior tibial artery pseudoaneurysm,” “chronic pseudoaneurysm with bone erosion,” and “intraosseous pseudoaneurysm extension.” Less than 40 cases of posterior tibial artery pseudoaneurysm were identified, and none clearly described transcortical erosion.

Here, we present a case of posterior tibial artery pseudoaneurysm causing tibial transcortical erosion, emphasizing the multimodality imaging finding and key differentiating points from soft tissue or osseous pathology.

## 2. Clinical Presentation

A 33‐year‐old male patient with no known medical illness presented with progressively enlarging swelling over the left anterior proximal shin of 1‐year duration. The swelling is associated with knee stiffness but no claudication or weight loss. He had a history of gunshot trauma to the left posterior knee soft tissue 2 years prior, which was managed conservatively. Based on the available medical records, no bone fracture or joint dislocation was documented, and no surgery was performed. The wound healed without immediate complications.

The patient has no known history of deep venous thrombosis, history of travel, or prolonged immobilization. Basic laboratory workup including CBC, CRP, and coagulation profile (PT, PTT, and INR) were within normal range.

On physical examination at presentation to an orthopedic specialty center, a small swelling was noted over the anterior aspect of the proximal left leg. The lesion was partially compressible and mildly tender. It was nonerythematous, with no overlying ulceration or sinus formation. Pulsatility was not clearly appreciated on initial examination. No audible bruit was documented. Distal dorsalis pedis and posterior tibial pulses were palpable and symmetrical.

Because the lesion was located adjacent to the tibial cortex and far from major vascular structures, a possibility of primary bone tumor with soft tissue component (including aggressive entities such as osteosarcoma), metastatic lesion, or soft tissue sarcoma was initially considered in the differential diagnosis. The patient was directly evaluated by orthopedic specialists, and based on the clinical suspicion of a neoplastic process, fine‐needle aspiration (FNA) was attempted prior to imaging assessment, which resulted in significant bleeding, and the procedure was terminated. Hemostasis was achieved by direct compression and pressure dressing; no tourniquet was applied. The patient remained hemodynamically stable. Then, ultrasound and MRI were performed, followed by CTA for further characterization.

## 3. Imaging Findings

### 3.1. Ultrasound Finding

Ultrasound examination demonstrated a large anechoic cystic mass with peripheral hypoechoic solid components in the left popliteal fossa, extending inferiorly into the proximal leg, deep to the soleus muscle. The lesion measured 7.7 × 4 cm (Figure 1A). On color Doppler examination, a yin–yang flow pattern was noted within the cystic component, and communication with the normal sized posterior tibial artery was appreciated (Figure 1B). Additionally, an anteromedial tibial cortical breach was noted, with a 2.8 × 7.8 − cm cystic lesion having communication with the medullary cavity (Figure 2A). Spectral Doppler showed bidirectional (to‐and‐fro waveform) flow (Figure 2B).

**Figure 1:**
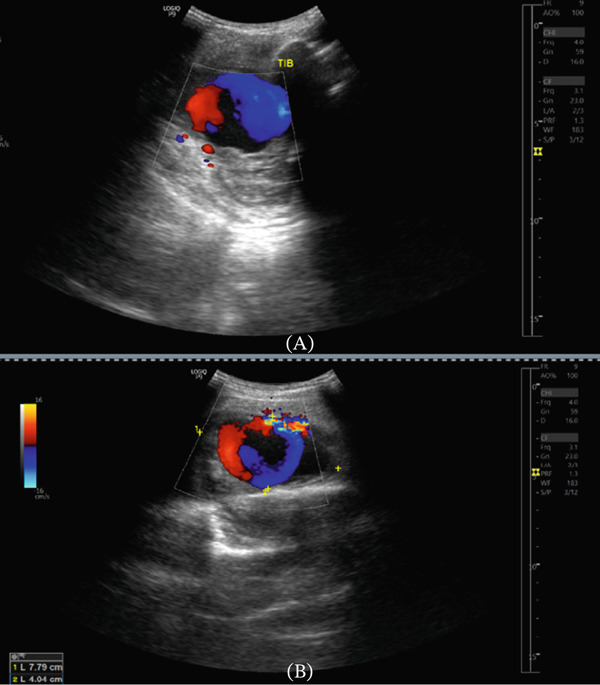
Sonographic images through posterior left knee show large cystic mass with peripheral solid echogenic content suggestive of thrombosis. (A, B) On color Doppler study, only the anechoic portion demonstrates flow with yin–yang pattern is noted.

**Figure 2:**
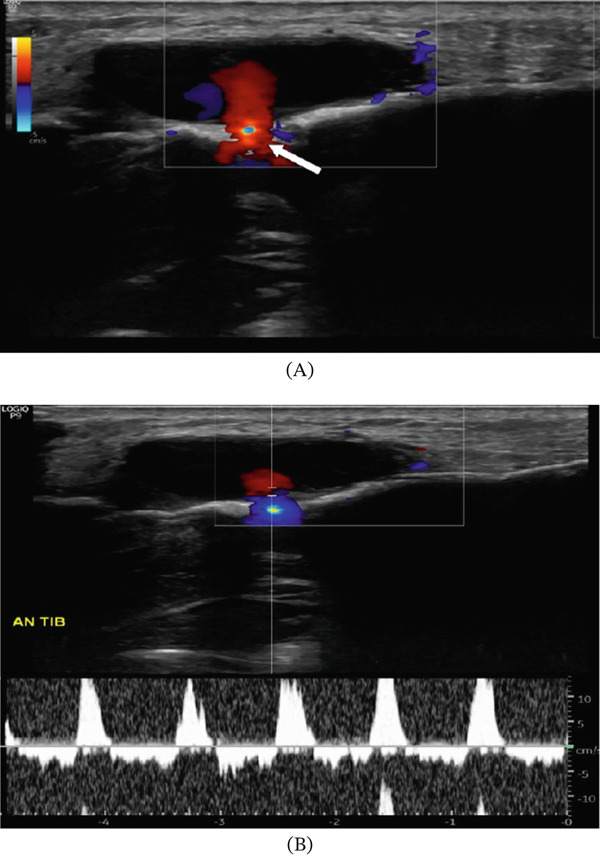
Color Doppler sonographic image through the left shin demonstrate a small anechoic cystic lesion adjacent to the anterior tibial cortex with a prominent color flow‐through a focal cortical defect (white arrow on Panel A). (B) On spectral Doppler study, bidirectional (to‐and‐fro) flow pattern is noted.

### 3.2. MRI Finding

Contrast‐enhanced MRI of the left leg demonstrated a well‐defined expansile popliteal fossa mass with transcortical extension into the tibia via a posterior cortical defect (Figure 3A). It showed heterogeneous hyperintense signal on T1‐weighted (Figure 3B) and PD fat‐saturated sequences (Figure 3C), with no significant postcontrast enhancement. The lesion further extends through an anterior cortical breach into the superficial subcutaneous soft tissues (Figure 4).

**Figure 3:**
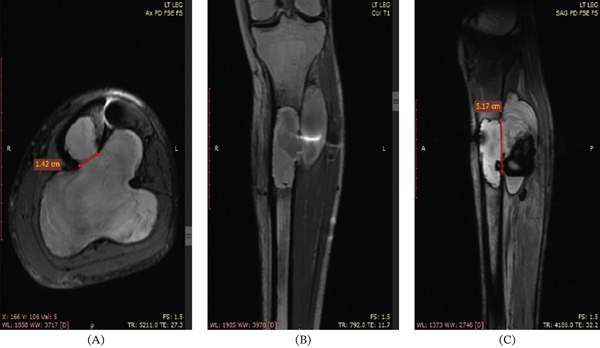
(A) Axial PD fat sat, (B) coronal T1, and (C) sagittal PD fat sat; MR sequences show a large heterogeneously hyperintense mass in the posterior proximal leg, which has clear tibial bone intramedullary extension through a large posterior cortical breach (measuring 5.17 × 1.42 cm). Susceptibility artifacts are noted in three images (likely from bullet fragments which are shown on the CT image of Figure 5).

**Figure 4:**
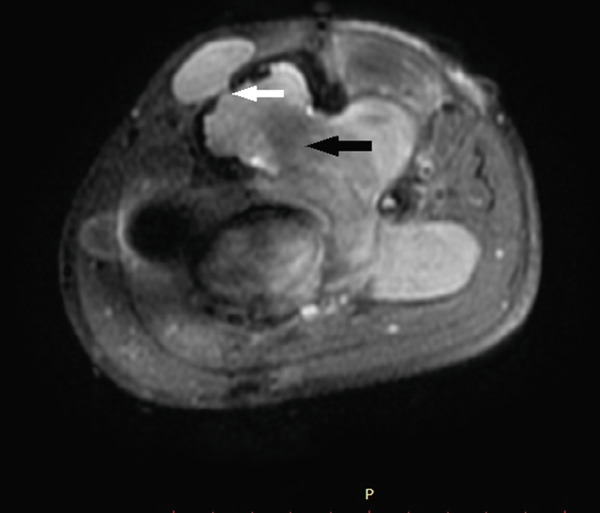
Axial T1, fat sat, and postcontrast images at proximal tibia show transcortical erosion with the lesion extending through a large posterior (black arrow) cortical defect and into the anterior shin through a smaller defect (white arrow).

### 3.3. CTA Finding

Follow up CTA of the lower extremity which in turn showed large outpouching from the proximal posterior tibial artery with the neck measuring 9 mm (Figure 6A,B). The sac demonstrated peripheral thrombus with an enhancing central lumen (Figure 6B). No active extravasation was seen at the time of imaging. Distal runoff was preserved with normal opacification of distal posterior tibial branches. A large defect was seen in the posterior aspect of the tibia with the thrombosed portion of the vascular lesion extending into the medullary cavity then into anterior subcutaneous tissue through a small defect (Figure 7A–C).

Axial CTA bone‐window image of the left leg demonstrates a small metallic density foreign body within the soft tissues, likely representing a retained bullet fragment from prior gunshot injury (Figure 5).

**Figure 5:**
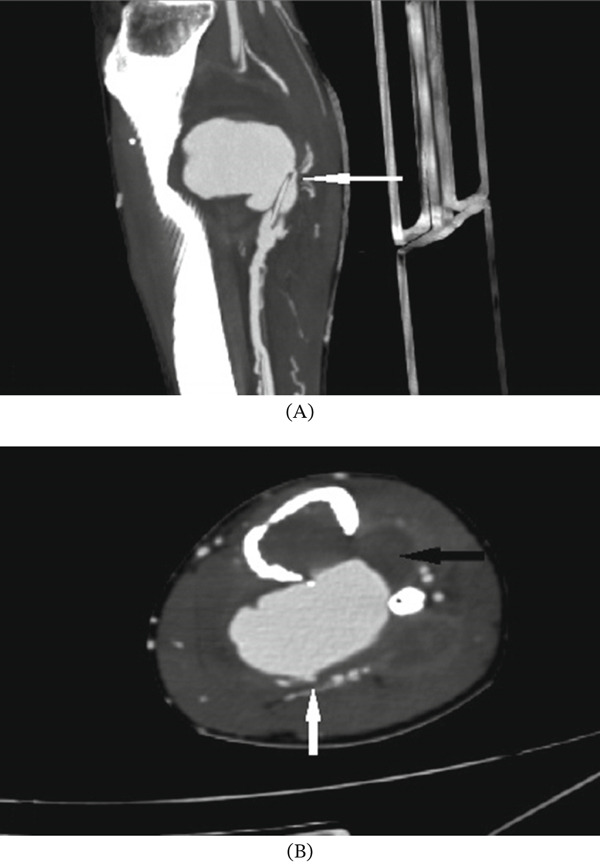
(A) Sagittal oblique and (B) axial CT angiography of the left leg shows a large contrast opacifying lesion, which has a small direct communication with the posterior tibial artery (white arrow, on both images). The lesion also contains a hypodense nonenhancing periphery with lack of contrast filling (black arrow), which is suggestive of a thrombus.

**Figure 6:**
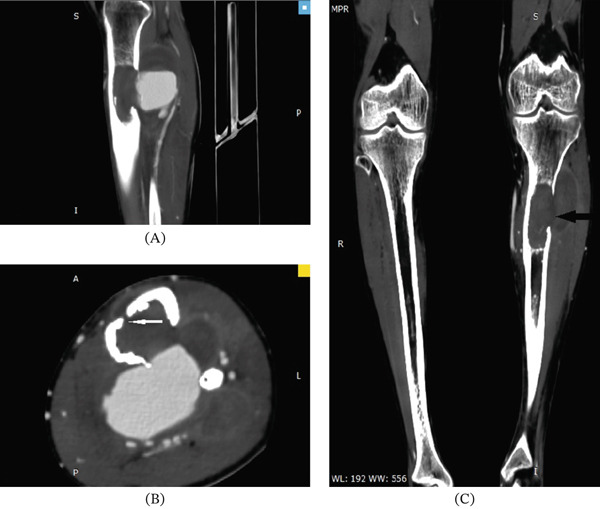
(A) Sagittal, (B) axial, and (C) coronal CT angiography images showing the thrombosed segment of the pseudoaneurysm within the medullary cavity, which then extends into anterior subcutaneous tissue through a small anterior tibial cortical defect (white arrow, Pannel B). The large posterior cortical defect, along with the thrombosed intramedullary segment of the lesion, is well depicted (black arrow, Pannel C).

**Figure 7:**
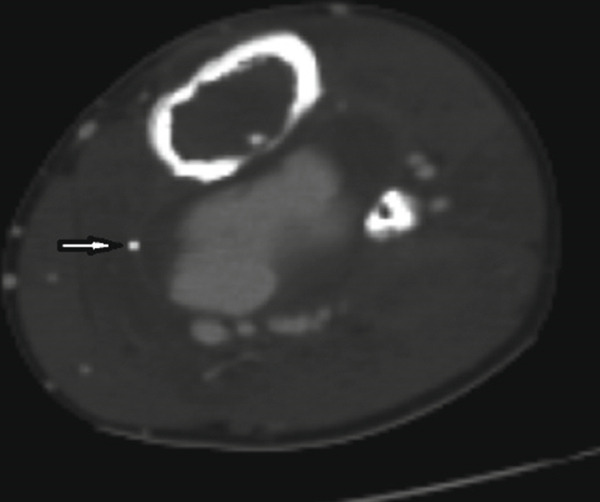
Axial left leg CT angiography in bone window shows small metallic density foreign body (white arrow), likely a bullet fragment from prior gunshot injury.

In summary, the presence of yin–yang sign as well as to‐and‐fro waveform on Doppler study, enhancing sac with neck on CTA, and direct arterial communication on both imaging modalities are important findings to differentiate a vascular lesion from soft tissue or an aggressive bone tumor.

## 4. Management

Due to the advanced nature of the pseudoaneurysm, surgical management was undertaken. Postprocedure, distal posterior tibial artery pulsation was restored. At 1‐month follow‐up, there was complete resolution of swelling with intact distal pulses and no neurological deficit.

## 5. Discussion

Posterior tibial artery pseudoaneurysms are extremely rare with less than 30 reported cases published in the literature [2]. They typically arise from penetrating or blunt trauma, lower limb fractures, and orthopedic interventions [2–6, 11]. Notably, our case is a result of a penetrating bullet injury, fitting one of these established causes.

Although often initially asymptomatic and found incidentally during imaging or surgery, pseudoaneurysms typically develop symptoms as they enlarge. Symptomatic cases commonly present with local or systemic signs like pain, swelling, and an expanding pulsatile mass, with symptom severity linked to size [3, 12]. Compression of the adjacent nerves or veins by a pseudoaneurysm can result in neuropathy, venous obstruction, and even thrombosis. Additionally, a ruptured pseudoaneurysm may lead to compartment syndrome, which is associated with high rates of morbidity. If left untreated, posterior tibial artery pseudoaneurysms can lead to vascular rupture, limb ischemia, and in severe cases, limb loss or rarely death [6, 7, 13–15]. Incorporating imaging early in the assessment of masses after trauma can prevent iatrogenic rupture and significant bleeding, a complication encountered in our case.

Doppler ultrasound is the first‐line imaging modality for evaluation of pseudoaneurysms, giving detailed information on the size, flow pattern, dimension of the neck, and feeding vessel. It characteristically shows turbulent flow with the “Yin‐yang” or “Pepsi” sign on color Doppler and a “to‐and‐fro” waveform in the neck on spectral Doppler analysis; both of these were demonstrated in our case. CTA shows a contrast‐filled sac, usually with an area of low attenuation indicating possible thrombosis, and shows the pedicle artery and its relation to the sac. MR angiography can be performed in patients in whom iodinated contrast or radiation is not appropriate. Additional MRI features supportive of a vascular etiology include flow‐related signal voids and pulsation artifacts. Conventional angiography remains the gold standard, with real‐time hemodynamic information, and is particularly helpful when the diagnosis is uncertain [3, 9, 16].

The complication of tibial transcortical bone erosion observed in this case is exceptionally unusual. However, similar erosive changes have been reported in other anatomical locations, such as vertebral bone erosion secondary to contained ruptures of abdominal aortic aneurysms. The proposed likely mechanism involves the progressive expansion of the cystic mass and repetitive mechanical trauma from the pulsatile nature of the pseudoaneurysm. In such cases, bone erosion is also considered indicative of the chronicity of the aneurysm [17, 18]. Our case presents a chronic course, and the mechanisms discussed above may plausibly explain the large tibial erosions.

A soft tissue mass with associated osseous involvement due to a pseudoaneurysm may mimic a tumor, raising concern for a locally aggressive primary bone tumor or a soft tissue neoplasm with secondary bone extension. However, the absence of a solid enhancing mass and lack of aggressive periosteal reaction and vascular encasement instead of arterial communication make a neoplastic process less likely [8, 16, 19].

### 5.1. Teaching Points

From this study, we have identified the following teaching points:

• Pseudoaneurysm can mimic an aggressive bone or soft tissue tumor, particularly when associated with cortical erosion and intraosseous extension.
• Doppler ultrasound demonstrates characteristic yin–yang color flow and to‐and‐fro spectral waveform, confirming vascular origin.
• CTA shows arterial phase enhancement with direct communication to the parent artery.
• MRI may demonstrate flow voids and pulsation artifacts, supporting a vascular etiology.
• Biopsy should be avoided prior to imaging confirmation due to risk of catastrophic hemorrhage.

## 6. Conclusion

Although rare, posterior tibial artery pseudoaneurysms should be considered in the differential diagnosis of calf lesions. Doppler ultrasound, CTA, and MRI play a crucial role in establishing the diagnosis. This case also highlights the importance of appropriate imaging evaluation prior to tissue sampling, as biopsy of vascular lesions such as pseudoaneurysms may result in catastrophic hemorrhage.

**Nomenclature**
CT: computed tomography
MRI: magnetic resonance imaging
US: ultrasound
FNA: fine‐needle aspiration

## Funding

No funding was received for this manuscript.

## Ethics Statement

Written informed consent was obtained from the patient for publication of this case report and accompanying images.

## Conflicts of Interest

The authors declare no conflicts of interest.

## Supporting information


**Supporting Information** Additional supporting information can be found online in the Supporting Information section. Filed CARE checklist including patient timeline, diagnostic reasoning, differential diagnosis, intervention, and follow‐up outcomes has been prepared and is submitted as Supporting Information.

## Data Availability

The data that support the findings of this study are available on request from the corresponding author. The data are not publicly available due to privacy or ethical restrictions.
