# Supplementary material for: Tibial Bone Transcortical Erosion Caused by a Posterior Tibial Artery Pseudoaneurysm: A Rare Case Report
Source: Case Rep Radiol. 2026 Jun 8;2026:1862171. doi: 10.1155/crra/1862171 (PMC13247140; doi:10.1155/crra/1862171)
Supplement: Supplementary file 1 — Supporting Information Additional supporting information can be found online in the Supporting Information section. Filed CARE checklist including patient timeline, diagnostic reasoning, differential diagnosis, intervention, and follow‐up outcomes has been prepared and is submitted as Supporting Information. [file CRRA-2026-1862171-s001.docx]

**CARE Checklist**

**Title:** Tibial Bone Trans-Cortical Erosion Caused by a Posterior Tibial Artery Pseudoaneurysm: A Rare Case Report

**1. Title**

- The diagnosis is included and it states this is a case report. It also specifically mentions the unique feature which is trans cortical erosion

**2. Keywords**

- Important key words for MESH are added likely Aneurysm, Pseudoaneurysm and Posterior tibial artery

**3. Abstract**

- Proper background, Case presentation, followed by about the imaging modalities, management and conclusion highlighting clinical significance were included

**4. Introduction**

- In this segment information about pseudoaneurysms, specifically focusing on posterior tibial artery involvement, and the lack of full documented trans cortical erosion were added.

**5. Patient Information**

- Our patient is adult male (33-year-old) with no known medical illness. He sustained a remote gunshot injury to left posterior knee (2 years prior) soft tissue which was managed conservatively. And currently he presented with a progressive left anterior shin swelling for 1 year

**6. Clinical Findings**

- Initial physical examination shows partially compressible swelling over left proximal tibia. The presence of pulsatility was not clearly appreciated initially. No other abnormalities seen in the left limb. Basic laboratory tests were unremarkable. Following FNA attempt, he had bleeding but controlled with compression alone

**7. Timeline summary**

| **Time** | **Event** |
| --- | --- |
| 2 years prior | Gunshot injury to left posterior knee soft tissue managed conservatively |
| For the past 1-year | Progressive anterior shin swelling |
| At presentation | Orthopedic evaluation; FNA attempted which resulted bleeding |
| Same admission | Doppler ultrasound performed |
| Subsequently | MRI (contrast-enhanced) |
| Final imaging | CT angiography better describe and confirms the case of pseudoaneurysm |
| Treatment | Open surgical repair |
| 1-month clinical follow-up | Resolution of swelling, intact pulses |

**8. Discussion**

- Focused on the trans cortical erosion as rare pseudoaneurysm complication
- Tried to address the importance of imaging before any invasive procedures
- The general expected complication from the pseudoaneurysm
- Comparison with similar literature

**9. Informed Consent**

- Written informed consent was obtained from the patient for publication of this case report and accompanying images
